# Comparison of Different Types of Palatal Expanders: Scoping Review

**DOI:** 10.3390/children10071258

**Published:** 2023-07-21

**Authors:** Angelo Michele Inchingolo, Assunta Patano, Matteo De Santis, Gaetano Del Vecchio, Laura Ferrante, Roberta Morolla, Carmela Pezzolla, Roberta Sardano, Leonardo Dongiovanni, Francesco Inchingolo, Ioana Roxana Bordea, Andrea Palermo, Alessio Danilo Inchingolo, Gianna Dipalma

**Affiliations:** 1 Department of Interdisciplinary Medicine, University of Bari “Aldo Moro”, 70121 Bari, Italy; angeloinchingolo@gmail.com (A.M.I.); assuntapatano@gmail.com (A.P.); matteo_des@hotmail.it (M.D.S.); dr.gdelvecchio@gmail.com (G.D.V.); lauraferrante79@virgilio.it (L.F.); robertamorolla@gmail.com (R.M.); c.pezzolla3@studenti.uniba.it (C.P.); robertasardano@gmail.com (R.S.); leonardodng1994@gmail.com (L.D.); ad.inchingolo@libero.it (A.D.I.); giannadipalma@tiscali.it (G.D.); 2 Department of Oral Rehabilitation, Faculty of Dentistry, Iuliu Hatieganu University of Medicine and Pharmacy, 400012 Cluj-Napoca, Romania; 3 College of Medicine and Dentistry, Birmingham B4 6BN, UK; andrea.palermo2004@libero.it

**Keywords:** rapid palatal expander, hyrax, leaf expander, rep on screw, slow maxillary expansion, palatine suture, dental anchorage, leaf spring activated expander, transverse maxillary deficit

## Abstract

Maxillary bone contraction is caused by genetics or ambiental factors and is often accompanied by dental crowding, with the possibility of canine inclusion, crossbite, class II and III malocclusion, temporomandibular joint disorder, and obstructive sleep apnea (OSAS). Transverse maxillary deficits, in which the maxillary growth is unusually modest, are frequently treated with maxillary expansion. The purpose of this study is to compare the dental and skeletal effects of different types of expanders, particularly the Leaf Expander, rapid and slow dental-anchored or skeletal-anchored maxillary expanders. Methods: We chose studies that compared effects determined by palatal expansion using a rapid palatal expander, expander on palatal screws, and leaf expander. Results: Reports assessed for eligibility are 26 and the reports excluded were 11. A final number of 15 studies were included in the review for qualitative analysis. Conclusions: Clinically and radiographically, the outcomes are similar to those obtained with RME and SME appliances; Therefore, it might be a useful treatment choice as an alternative to RME/SME equipment in cases of poor patient compliance or specific situations. Finally, all of the devices studied produce meaningful skeletal growth of the palate. The use of skeletally anchored devices does, without a doubt, promote larger and more successful growth in adolescent patients.

## 1. Introduction

Transverse maxillary deficits, which are characterized by abnormally low maxillary growth, are frequently treated with a rapid maxillary expansion (RME) [1,2,3]. A total of 8–22% of orthodontic patients have this transverse maxillary discrepancy [4,5]. This condition’s etiological reasons may be inherited or ambiental, and it is often accompanied by dental crowding, with a possible risk of canine inclusion, crossbite, class II and III malocclusion, dysfunction of the TMJ, and obstructive sleep apneas (OSAS) [6,7,8]. Regardless of surgical expansion, RME in orthodontics is typically carried out utilizing the RME hyrax appliance, leaf expander appliance, and MARPE appliance. The palatine suture opens up and the palate widens as a result of the various activation regimens of these appliances. From the anterior palatine fissure to the posterior nasal spine, the median palatine suture runs [9,10]. As the patient grows, more spicules form along the suture, resulting in stringent interdigitations. The ossification process of the suture is directly associated with skeletal age and gender [11,12,13]. It gets harder to open the suture as the patient ages. Research examines six cervical vertebral maturation stages, examining their association with peak stature growth [14,15,16]. Between cervical vertebral stages 3 and 4, mandibular and craniofacial growth increases, with rectangular bodies and concavity on lower edges [16,17]. Angelieri et al. uses CBCT examination to examine palatine suture morphology in five stages, showing partially interdigitated lines with high-density lines and low bone density areas [14].

Therefore, the most precise method for assessing the palatine suture and detecting dentoskeletal alterations following RME is CBCT. Cone-beam computed tomography (CBCT) can be used to evaluate the palatine suture bone bridges’ interdigitation quality and density, which is a crucial factor in the device selection process [6,7,18].

The most contentious choice in treating transverse disparities in adolescent patients is how to expand the upper jaw. Depending on the situation, the orthodontist should select a “patient-oriented” appliance that can reduce the variety of potential side effects, including appliance breakage, functional issues, periodontal tissue problems, and discomfort [19]. The hyrax expander (HEX), a permanent appliance with no externally visible elements that are fastened to the upper first molars (to deciduous teeth when possible), is the most widely used palatal expander [8,20,21,22]. It has a central screw that, when turned as directed by the orthodontist (about 0.25 mm twice daily for 15 days), applies a force of around 10 kg to split the palatine suture, causing the transverse diameter of the palate to enlarge. Thus, the suture can be divided in a short period of time—in most situations, it takes around 15 days to do this [7,23,24]. After this, the expander must be left in place for an additional six to nine months to allow the suture to ossify and consolidate in the new spaces [25].

Next to the conventional HEX, the Leaf expander (LEX) has been proposed as an alternative without parental cooperation [26]. The patient should experience substantially less discomfort as a result. Similar to HEX, it comprises a metal skeleton with two bands that are bonded to the molars (deciduous wherever possible), as well as an expansion screw that makes use of mild continuous forces produced by Ni-Ti springs. Comparing LEX to HEX, the expansion takes place over a longer period of time. Along with the vascular growth that promotes rapid neo-ossification, the intermaxillary suture mineralizes concurrently with this gradual expansion, making it more consistent with histophysiology. The Leaf expander (LEX) has been suggested as an alternative to the traditional HEX without parental consent. The patient should experience substantially less discomfort as a result [27]. Similar to HEX, it comprises a metal skeleton with two bands that are bonded to the molars (deciduous wherever possible), as well as an expansion screw that makes use of mild continuous forces produced by Ni-Ti springs. Compared to HEX, LEX has a different action kinetics and the growth continued for several months [28]. It is more consistent with histophysiology during this sluggish growth because the intermaxillary suture is mineralized concurrently with the expansion and because vascular development encourages rapid neo-ossification [29].

While HEX and LEX expanders are intended to act orthopedically, they invariably result in unintended tooth movements. These tooth motions have the potential to change how well the teeth and their roots are supported by the periodontal ligaments, but they can also create space. From a biological perspective, the prognosis is really uncertain when such extension is sought in adult patients or those who have otherwise reached their maximal growth because of the increased interdigitation of the maxillary sutures and the rigidity of the surrounding structures.

Rapid palatal expansion by skeletal anchoring (MARPE) can be employed on patients who are in advanced phases of skeletal development to avoid unfavorable dentoalveolar consequences and maximize skeletal expansion potential [3,26]. Lee et al. proposed the first report of RME with skeletal anchoring in 2010 [30]. Two tiny implants in the palate were used in the trial on a 14-year-old girl. The maxillary molars showed a modest buccal inclination and successfully separated the mid-palatal suture. A second research using MARPE on 69 adult patients with an average age of 20.9 years revealed an opening of the mid-palatal suture success rate of 86.96% and an average increase in maxillary breadth of 2.11 mm. Additionally, there was a noticeable rise in the inter-molar distance and nasal cavity width, measuring 1.07 mm and 8.32 mm, respectively [31]. The MARPE, therefore, appears to be a viable alternative in cases of young adults, subject of course to a good diagnostic assessment [32,33].

The purpose of this study is to compare the dental and skeletal effects of different types of expanders, particularly the Leaf Expander, rapid and slow maxillary expanders with dental or skeletal anchorage to determine whether there is one method that is more effective and efficient than the others and to evaluate the pain they cause patients.

## 2. Materials and Methods

### 2.1. Protocol and Registration

This study was carried out in accordance with the Preferred Reporting Items for Systematic Reviews and Meta-Analyses Extension for Scoping Reviews (PRISMA-ScR) guidelines and submitted to PROSPERO (International Prospective Register of Systematic Reviews) with ID 430622 [34,35,36,37].

### 2.2. Search Processing

With a restriction on English-language studies published between 1 January 2013 and 13 April 2023, we searched PubMed, Scopus, and Web of Science. To undertake an evaluation that is current with the recent 10 years, this time frame was selected. The search approach included the following Boolean keywords: (“hyrax” OR “rep on screws” OR “leaf expander”) AND “treatment”. These phrases were chosen because they most accurately reflected the aim of our investigation, which was to find out more about the expansion brought about by employing various palatal expanders.

### 2.3. Eligibility Criteria and Study Selection

A quick palatal expander, an expander on palatal screws, and a leaf expander (Leaf Expander^®^, Leone Ortodonzia e Implantologia, Via del Ponte di Quaracchi, 50, 50019 Sesto Fiorentino FI) were used in the research and we chose to compare the effects of palatal expansion. The two steps of the selection process were the appraisal of the title and abstract and the complete text. Any article that fit the following requirements was taken into consideration: (a) clinical trials including human intervention; (b) comparison of the therapy to other interventions; (c) English language complete text. Publications (such as meta-analyses, research methods, conference papers, in vitro, or animal experiments) that lacked original data were not included. Titles and abstracts from the preliminary search were retrieved and evaluated for relevance. Full articles from pertinent research were acquired for further analysis. The retrieved studies were assessed for inclusion using the aforementioned criteria by two different reviewers (R.M. and A.P.).

### 2.4. Data Processing

R.M. and A.P., two reviewers, independently evaluated the studies’ quality based on selection criteria after doing a database search to extrapolate the findings. To use with Zotero, the chosen articles were downloaded in the 6.0.15 version. A senior reviewer (F.I.) was consulted in order to address any disagreements between the two writers.

### 2.5. PICOS Requirements

The PICOS (Population, Intervention, Comparison, Outcome, and Study Design) criteria, which are used in this evaluation, encompass population, intervention, comparison, outcomes, and study design (Table 1).

## 3. Results

### Selection and Characteristics of the Study

**Figure 1:**
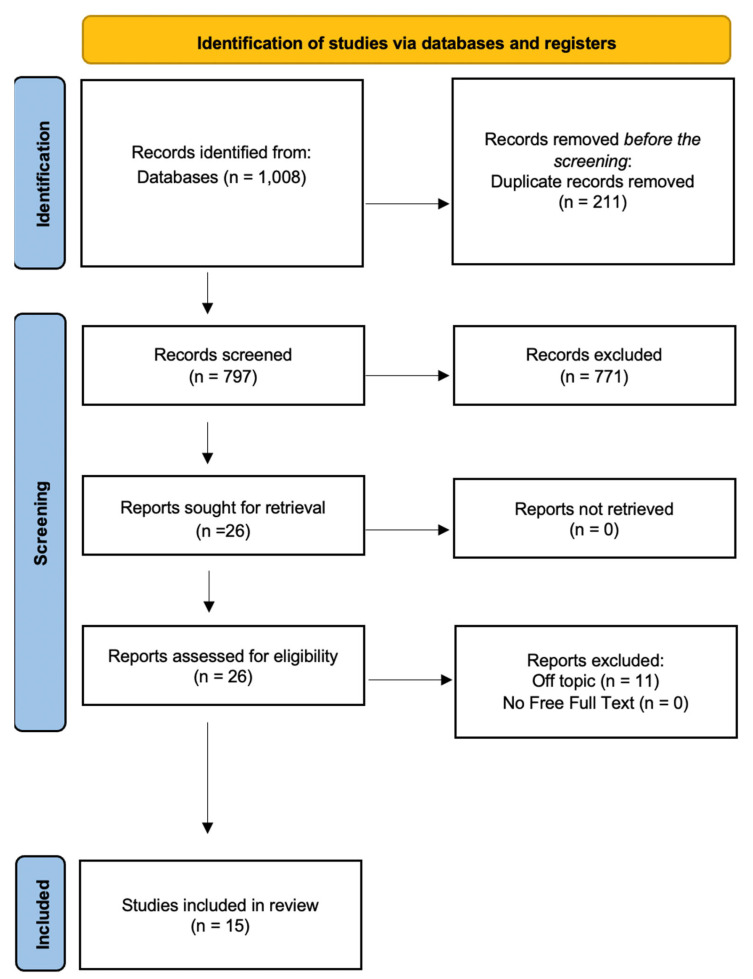
PRISMA ScR flowchart diagram of the inclusion process.

A total of 1008 publications were found in the online database (PubMed *n* = 675, Scopus *n* = 1, and Web of Science *n* = 332); no papers were found using a manual search. After 211 duplicate studies were removed, 797 studies were evaluated by looking at the title and abstract. A total of 26 records were chosen out of 771 items that failed to fulfill the requirements for inclusion. Reports requested for retrieval totaled 30, while reports not found totaled 0. There were 26 reports evaluated for eligibility, and 11 reports were removed. In the end, 15 studies were reviewed for the qualitative analysis. The selection process and the summary of selected records are shown in Figure 1. The studies characteristics are summarized in Table 2.

## 4. Discussion

Upper jaw transverse deficit associated with posterior crossbite is one of the most prevalent malocclusions in developing patients, affecting an estimated 13.3% of adolescents [41].

The transversal maxillary deficit does not resolve spontaneously with growth, therefore, it is advisable to intervene early to resolve it, thus preparing the bone bases for a correct eruption of permanent teeth [49,51].

In 1860, Angell invented the first maxillary expander device (RPE) [47].

It was immediately considered safe and efficient and met with great success [49].

Since then, the interest in the RPE has gradually increased.

**Figure 2:**
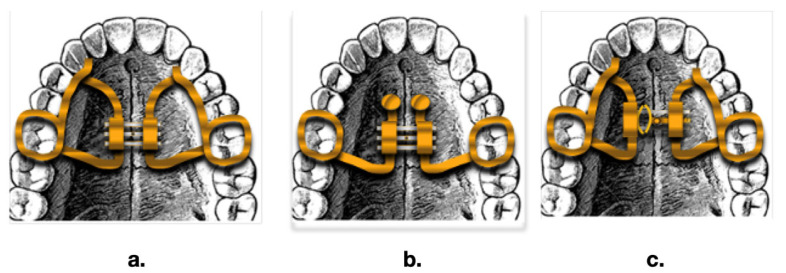
Three different types of palatal expander: (**a**) Hyrax expander; (**b**) Rep on mini screws; (**c**) Leaf expander.

Maxillary expansion can be achieved with slow (SME) or rapid (RME) expanding devices. RME is typically the first-choice treatment for transverse skeletal discrepancies [38] (Figure 2).

### 4.1. Effects of Leaf Expander

#### 4.1.1. Dental Changes

Manzella et al., 2017 conducted a retrospective controlled clinical study to analyze how the Ni-Ti Memoria^®^ Leaf Spring Activated Expander (MLSAE) influences dental health in young patients with orthodontics who have reduced transverse dimensions of the upper jaw. The Ni-Ti MLSAE was used by 22 patients (mean age of 12 years) compared to 22 untreated controls. Digital dental casts were taken at the pretreatment, one-week, monthly, and post-expansion time periods. The maxillary dental arch’s inter-canine, inter-premolar, inter-first molar, arch depth, arch perimeter, and molar angulation measures were evaluated [44]. The overall mean expansion time was 4.21 months. Significant increases were seen in the treatment group’s inter-canine, inter-first and second premolar, inter-first molar, and arch perimeter measures between the first measurements and the final ones. There were no observable differences between the controls. According to between-group studies, there were statistically significant differences in all variables between the treatment and control groups, with the exception of arch depth and molar angulation. Average changes were 1.04, 5.65, 5.80, 4.70, and 2.15 mm for inter-canine, inter-first premolar, inter-second premolar, inter-first molar, and arch perimeter, respectively. The Ni-Ti MLSAE can achieve sufficient expansion in patients between the ages of 6 and 16 without significantly tipping the teeth. When using the recommended protocol, it should be regarded as a device for slow expansion that enables measured expansion at an average rate of 1.1–1.5 mm per month [44].

**Figure 3:**
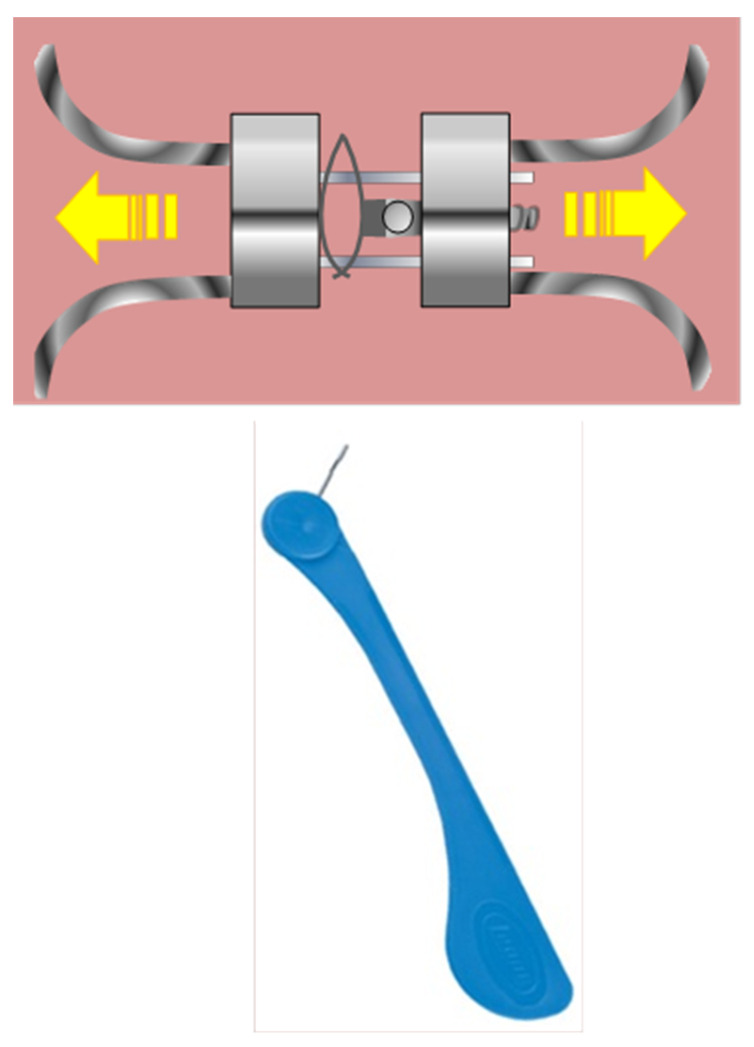
Leaf expander with key activator.

Given its potential to bring about positive anatomical and functional changes, the Leaf Expander (Figure 3) may be regarded as the first therapeutic option for treating mast cell tumors. The primary benefits of using this device lie in its simplicity of activation, lack of cooperation (no compliance), and the ability to control the movement of the teeth using light, predictable, and cost-effective forces. The findings of a study conducted by Lanteri et al. in 2018 demonstrate the effectiveness and usefulness of the LE even when applied to solid teeth for the correction of transverse maxillary deficit in growing populations. Complete correction of the posterior crossbite occurred in all patients in an average of 4 months, with a natural expansion of the first permanent molars. They were obtained [25].

In 2020, Cossellu and others said that Leaf Expander therapy is an effective therapeutic option for treating transverse maxillary deficit [49].

#### 4.1.2. Alterations of the Buccal Alveolar Bone of the Permanent First Molars and the Deciduous Second Molars

Lanteri et al. 2020 conducted an interesting clinical study. After maxillary extension employing a gradual expansion protocol of the maxilla, the alteration of the vestibular bone thickness and vestibular tipping of primary second molars and permanent first molars were investigated. With a mean age of 7 years, the Leaf Expander procedure was applied to nine males and eleven women. Cone beam computed tomography (CBCT) images were used to calculate the BT, BH, interdental angle (TIP), and IW of the first and second primary molars. The bone width from the vestibule to the second primary molar and the intermolar size of both teeth were the only variables that statistically altered. It would appear that following treatment with LE, buccal bone depth from the vestibular to the first molars did not decrease significantly. Using a SME with Ni-Ti springs suggests to be a safe and effective approach for treating upper jaw hypoplasia during mixed dentition in clinical settings [45].

### 4.2. Comparison between RME and SME

#### 4.2.1. Comparison between Memory Screw and Traditional Hyrax Screw (Figure 4)

The memory screw, which has both screw and nickel-titanium springs, is the first banding device to apply a continuous force as opposed to intermittent force. The sagittal and vertical effects of maxillary expansion brought on by a memory screw (MS) on the dentofacial structures have not been investigated in any studies. The goal of Koray Halcolu and Brahim Yavuz’s study from 2020 was to examine the differences between the sagittal and vertical alterations in participants who had been treated with a memory screw with a traditional Hyrax screw (HS). Two groups of 32 patients with maxillary transverse deficit were created. A MSG included 17 patients (9f and 8m), while a HSG was composed of 15 people (8f and 7m). The individuals in the two groups were about 13 years old. In the early stages of therapy (T1), the end of the expansion phase (T2) and the end of the retention period (T3); lateral cephalograms of the patients were performed. In the MSG, the average duration of expansion was 7 days, whereas in the HSG, it was about 35 days. It may be said that all patients in both groups underwent sutural opening, which was followed by significant dental and bone development. In both groups, the mandible turned posteriorly and inferiorly as a result of the maxilla moving anteriorly and inferiorly. The MSG had a larger rotation. The newly created MS opens the mid-palatal suture and extends the maxilla with relatively lighter stresses over a shorter period of time, taking advantage of protocols for both quick and slow maxillary expansion [43].

**Figure 4:**
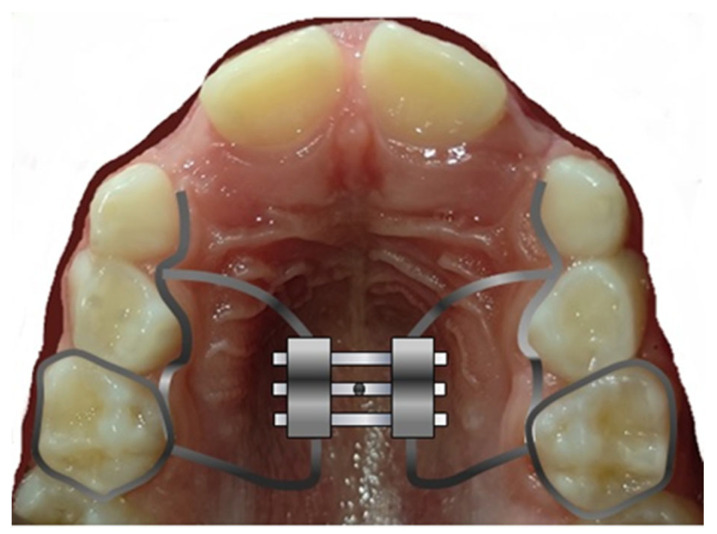
Hyrax expander on second primary molar.

#### 4.2.2. Dento-Skeletal Changes

Serafin et al. in 2022 conducted a randomized clinical in which Leaf Expander (LE) was used to compare RME and SME techniques [46]. Upper deciduous teeth serving as anchorage were analyzed with 3D CBCT before and after therapy. According to the current study’s findings, the following conclusions could be drawn: RME and SME can both produce bone and dentoalveolar transverse expansion equally well; the anchoring of deciduous teeth allows for clinically considerable transverse expansion while causing no injury to permanent teeth; patients with respiratory problems and anterior dental crowding may benefit more from RME, because it created a greater anterior expansion than SME; SME is advised for patients with posterior dental crowding. Because SME releases forces continuously, it also eliminates the need for home activation and allows a more controlled dental movement [46].

#### 4.2.3. Cephalometric Effects of Expansion

In 2018, Lanteri et al. also carried out a fascinating retrospective study. This study contrasts the dental and orthopedic effects of the LE with the results of fast and SME. Thirty patients with posterior crossbites were included in the sample and were split into three groups: RME (3m, 7f), with an average age of 8.9 years; SME with an average age of 12.2 years; LE, with an average age of 7.9 years. Postero-anterior cephalometric studies were taken before treatment started (T1) and nine months later (T2). A calibrated examiner has measured the width of the upper permanent molars, the mandible, the maxilla, and the nasal canal. After the treatment, every measurement went up significantly. Average maxillary length increased about by 4 mm, 2.8 mm, and 3.6 mm in the RME, SME, and LE groups, respectively. In the RME, SME, and LE groups, the width of the upper permanent molars increased by 5.4 mm, 5.4 mm, and 3.8 mm, respectively. There are no statistically significant differences between the groups. It has been proven that the LE is effective at correcting transversal deficiencies [25,52].

In 2021, Lanteri et al. performed retrospective research using bi-dimensional cephalometry to compare and contrast the skeletal and dental alterations brought on by the use of a RME and LE. Data were compared to a group that was not given any treatment. The LE group had an average age of 8 years and consisted of 11 females and 9 boys. A total of 11 men (mean age: 7 years) and 12 women (mean age: 8 years) made up the RME group in the current study. Dolphin Imaging software was used to trace digital cephalograms and calculate all reported measurements. No statistically significant change was discovered, with the exception of the angles between the upper incisor and the cranial base and bispinal planes, which showed a reduction, and the angles between the lower incisors and the mandibular plane, which showed an increase. It appears that both the RME and cause similar changes to the bones and teeth [38].

### 4.3. Comparison between RME Conventional Jaw Expander and Mini-Screw Supported Expander

**Figure 5:**
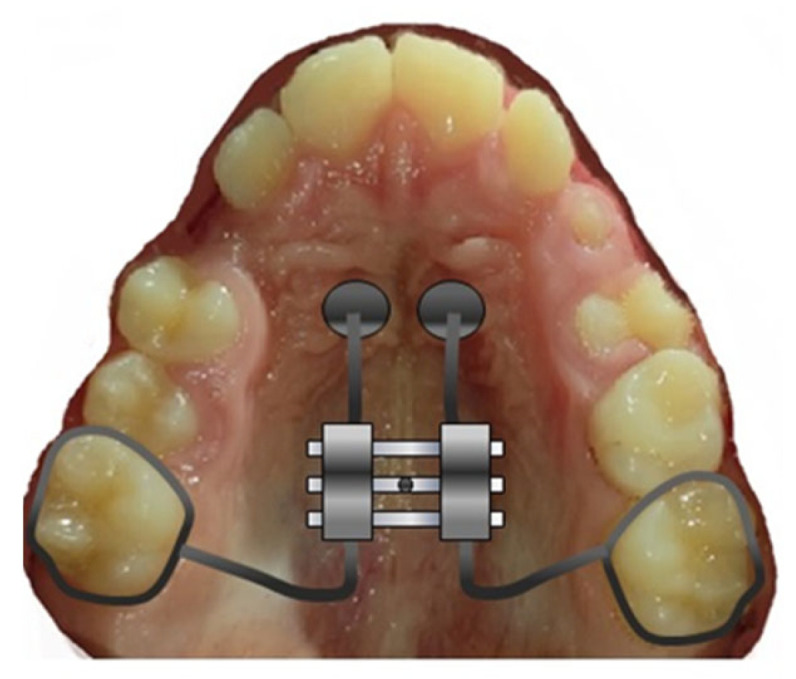
Bond anchored maxillary expansion with mini-screw.

In addition to the opening of the medio palatal suture, which is its main objective, RME causes various undesirable effects: buccal tipping, reduction of buccal bone and marginal bone levels, thinning of anchoring teeth, root resorption, crossbite recurrence, uncertain stability over time, and swelling and ulceration of soft tissues [53]. Many of these changes can be prevented by using mini-screws in the palatal bone (Figure 5) as an anchor in RME treatments [42].

Researchers have performed various studies over the years to increase the desired beneficial skeletal effects of palatal expansion and reduce its dental and tissue side effects [47].

For example, inserting four mini-screws into the Hyrax conventional expander design for the treatment of palatal bone contraction, prior to orthognathic surgery, has been shown to result in dental side effects and a high degree of stability over time. Other studies in this regard also allow us to conclude that expanders anchored with mini-screws to the bone can reduce the need for transverse orthognathic surgery [54].

Today, there are several types of bone-anchored jaw appliances. One of the first devices was the Dresden expander. The Dresden B-RME was used for the first time in Germany as a palatal distractor in adults undergoing orthognathic surgery, obtaining positive results in the bilateral symmetrical correction of the inverse bite.

The Dresden B-RME is supported by two bone anchors, a palatal implant on one side and a mini-screw on the other. For patient comfort and rapid insertion, the expander features two cylinders with three prongs for retention.

Tugce Celenk-Koca et al. in 2018 observed significant differences between two groups of adolescent patients undergoing RME, for all skeletal variables, except for the measurement of the width of the nasal cavity at the level of the maxillary first premolars [42,55].

- The Hyrax with dental anchoring supported the often-described wedge-shaped opening of the suture by showing the highest increase in width at the incisive foramen and the lowest rise at the level of the maxillary first molar.
- RME performed with anchorage on mini-screws showed a greater recovery of space in the premolar region and very similar increases in width in the incisive foramen and the molar suture. This led to a more parallel sutural opening with an almost three times greater expansion in the suture medio palatal [42]. The expansion, however, which took place with expanders on mini screws led to the straightening of the upper molars with a significant advantage for the buccal alveolar bone support.

There was no difference in the length of the molar roots between the two expansion groups, and most individuals in both groups had bigger anterior suture openings.

**Figure 6:**
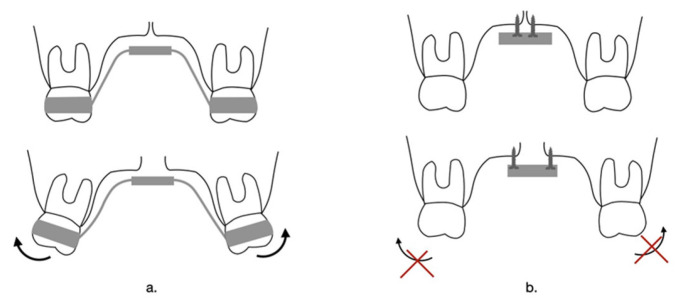
Dental effect of (**a**) Hyrax anchored on molars and (**b**) Rapid palatal expander anchored on mini screws.

An RCT research conducted in 2021 by Silveira et al. focused on palatal expansion by contrasting the dental outcomes and quality of life consequences of teenagers treated with Mini Hyrax and Hyrax expanders (Figure 6). With the help of a pre- and post-treatment and post-restraint questionnaire, the effect on quality of life was evaluated. Between the Mini Hyrax users and the Hyrax wearers, there were no appreciable variations in the dental impacts over time, as well as the influence on the quality of life: there was a worsening of function 14 days after the application of the devices probably determined by the activation of the screw and by the applied forces, while there was an improvement the overall quality of life six months after the start of treatment given by the adolescent’s recognition that he was on the way to resolving the malocclusion [48].

### 4.4. Evaluation of Skeletal Changes Using Three-Dimensional Images

As we know, before the introduction of three-dimensional (3D) imaging, such as conventional CBCT, to evaluate the results of orthodontic treatment with RPE on dentoalveolar structures, researchers have commonly used two-dimensional radiographic images such as lateral and posteroanterior cephalograms. However, these methods have many limitations and that is why today we prefer to use three-dimensional scans that allow us to measure, with values corresponding to reality, the skeletal, dental, dentoalveolar, and suture variations pre and post-expansive treatment [42,56].

The computerized cone-beam tomography was used in a study by Bruno de Paula Machado Pasqua et al. in 2022 to compare dental electrophysiological changes to rapid maxillary expansion with dental transmission equipment. The patient-accepted HH device divides the risk of expansion between two mini-screws in the front palate and two posterior teeth with orthodontic tubes. In addition, the risk of infection is low and the placement of mini-screws is minimally invasive. A total of 42 patients between the ages of 11 and 14 with a lack of transverse maxilla were divided into two study groups: HHG (Hybrid Hyrax group) and HG (Conventional Hyrax group) [41].

Before and three months following the activation period, the CBCT was conducted.

The following settings were used for the CBCT: 120 kVp, 18 mA, 8.9 s exposure time, 0.2 mm 25 voxel size, and a field of view (FOV) of 160 × 60 mm. Scans have been focused on the breast area to reduce radiation exposure to a minimum. To standardize the position of the test during CBCT acquisition, the patient was oriented with the Francoforte oblique piano parallel to the pavement and the Francoforte medial piano perpendicular to the latter. The scans were obtained prior to treatment (T0) and three months after the active phase (T1).

The Dolphin software was used to extract the data for analysis.

The primary objectives included changes to the nominal dimensions. The spacing between the teeth was used to measure dental changes in accordance with the points of the wearer’s cuspids and the tips of the palatal radicles. The axial inclinations (degrees) of maxillary first premolars and permanent first molars have been evaluated. All measurements were evaluated in the first premolar and first molar regions.

Dolphin software was used to extract data for analysis. First premolars and first permanent molars in the maxilla were measured for their axial inclinations (degrees). The first premolar and first molar areas served as evaluation sites for all measures.

In conclusion, Hybrid Hyrax has demonstrated more skeletal changes and less dental side effects, particularly in the area of the first premolar. The degree of activation has influenced the more significant scheletric nasal changes in the hybrid hyrax group.

- Compared to a dental expander (Hyrax), the osso expander (Hybrid Hyrax) has caused a greater increase in skeletal changes in the newborn cells’ primary region of the brain.

Hybrid Hyrax has shown a noticeably greater rise in the size of the nasal cavitation in the areas of the first premolars and the first molars; in the first premolars following RME with Hybrid Hyrax, only minimal changes in the angle of the teeth have been observed.

- There were no significant differences between the groups in terms of dental changes made after RME in the area of the first molars.
- The degree of activation has an impact on the more pronounceable scheletric nasal changes on Hybrid Hyrax.

These studies have several limitations that should be considered. For example, the generalizability of the results may be restricted to children between the ages of 11 and 14 as well as the types of technology used. These findings need to be carefully considered by patients outside of this age group.

### 4.5. Influence of Palatal Expansion on the Lower Arch

As argued by Cossellu et al. in 2020, based on some research carried out, the tongue plays a fundamental role during expansive orthodontic treatment, regardless of the type of device, because, due to the palatal presence of the expander, it lowers its position and together with the new intercuspidation, during chewing promotes expansive forces in the lower arch. The jaw expansion, therefore, acts on the balance of the tongue and cheek and consequently stimulates an expansive action in the lower arch [49].

### 4.6. Device’s Pain

The following table taken from Ugolini et al. study of 2020 shows the difference between the level and index of pain during the first four days after the use of two different devices: a leaf expander and standard Hyrax (Table 3) [20].

Pain caused by expansion is closely related to the activation protocol and lifestyle of the patient. The analysis was performed through patients’ completion of a questionnaire on the analysis of pain and discomfort caused by the apparatuses. For the analysis of pain intensity, the Wong–Baker scale was used, during the first week of apparatus placement. As shown in the table, it can be seen that subjects treated with leaf expander experienced less pain both from the point of view of intensity and duration than subjects treated with standard Hyrax [20].

In the following study of Nieri et al., 2021 the difference in pain generated in the first S12 weeks of treatment in leaf expander patients and conventional RME patients was analyzed. Patients and parents were asked to fill out a questionnaire, as a result of which the following results were obtained. Pain was analyzed according to the VAS scale, and it was reported that the subjects with conventional RME showed a higher mean of pain (0.6 ± 0.5), compared with the mean of the leaf expander-treated group (0.3 ± 0.4), and that the maximum difference in pain was noted mainly in the first week of treatment. Based on these data, we conclude that leaf expander causes fewer complications than conventional RME. The pain originated from the inflammation of the mediopalatine suture during its expansion and the pressure generated on the periodontal ligament, and it was shown that the pain fee decreased significantly with the use of ketoprofen or with the reduction of the number of daily apparatus activations [39,57].

In the following study, conducted by Feldmann et al., 2017, conventional hyrax and hybrid hyrax are compared, evaluating their pain at day 1 and day 4 after activation. There was not a statistically significant variation between the two groups, although hybrid hyrax showed slightly lower levels of pain, the only difference reported in the hyrax hibrid treated group compared to conventional hyrax was pain at the molars and incisors [50].

It was noted that patients treated with RME apparatology reported pain mainly during the first 10 activations, reaching a maximum level at day 3–4 and decreasing on subsequent days. Pain was analyzed with the VAS scale, and patients were asked to answer questions associated to jaw function impairment during free time, use of analgesics, overall discomfort, and pain severity [50].

## 5. Conclusions

After analyzing these studies, we can state that both rapid and slow expansion methodologies achieve similar expansion effects and, therefore, the choice of therapeutic means is individualized based on the patient’s degree of collaboration. The differences in expansion obtained between RME and Leaf expander are not clinically relevant. Therefore, the choice of the therapeutic means is the responsibility of the clinician.

In conclusion, all devices analyzed promote relevant skeletal expansion of the palate. Certainly, the use of skeletally anchored devices promotes greater and more effective expansion in the adult patient.

## Figures and Tables

**Table 1:** PICOS criteria.

Criteria	Application in the Present Study
Population	Children and adolescents (age range 6–16 years)
Intervention	Use of rapid palatal expander, rapid palatal expander on screws, leaf expander
Comparisons	Comparing before and after palate expansion with different types of palatal expanders.
Outcomes	Comparison of the palatal skeletal expansion techniques most used by ortho of different types of expanders and the pain they cause.
Study design	Clinical Trials.

**Table 2:** Characteristics of the studies included in the qualitative analysis.

Authors	Type of Study	Object	Study Design and Timeline	Results
Lanteri et al. (2021) [38]	Retrospective study	A comparison of cephalometric dental and skeletal alterations following the use of a RME and a Ni-Ti (Leaf) expander with a group of untreated controls.	Radiograms taken prior to and following maxillary growth were noted. There were 11 women and 9 men in the Leaf Expander group. There were 12 women and 11 men in the RME group.	Leaf expander and RME have overlapping effects in terms of dental and skeletal changes.
Nieri et al. (2021) [39]	Multicenter randomized controlled trial	Compare during the first 12 weeks the expansion determined by a screw with moderate and continuous forces against a screw for quick jaw expansion.	30 subjects in mixed dentition with a transverse discrepancy of at least 3 mm, with a posterior crossbite were divided into 3 groups: RME group; SME group; a leaf expander (LE) group. Comparison of these different techniques.	Major differences were found only in reference to algic sensitivity felt significantly less with leaf, particularly during the first 7 days. There were no differences detected for the other factors.
Lanteri et al. (2018) [40]	Retrospective study	Dental and skeletal comparison between the Leaf Expander and and slow and rapid maxillary expansion.	Nasal, maxillary, mandibular, and upper permanent molar width was measured in 30 patients with a posterior crossbite. They were divided into 3 treatment groups: (a) rapid maxillary expander (RME); (b) slow maxillary expander (SME); (c) leaf expander (LE).	No differences were found among the 3 groups, all measures increased significantly
De Paula Machado Pasqua et al. (2022) [41]	Randomized clinical trial	Comparison, through cone-beam computed tomography, the dento-skeletal changes obtained from the use of the Hyrax palatal expander and the Hybrid Hyrax.	42 patients divided into 2 groups: (1) group treated with Hyrax Hybrid (HHG); (2) group treated with Hyrax (HG). Before and 12 weeks after the activation phase, CBCT was performed, in order to evaluate the modifications.	The hybrid Hyrax had less dental effects than skeletal ones particularly in the first premolar area. In the HHG, the level of activation affected the greater nasal skeletal alterations.
Celenk-koca et al. (2018) [42]	Prospective randomized clinical trial	To assess the dental and skeletal changes in adolescents using conventional and miniscrew-supported maxillary expansion appliances.	40 patients divided into two groups: (1) rep with dental anchorage; (2) rep on miniscrews. Changes in transverse skeletal widths, buccal bone thickness, dental tip and root length were evaluated with cone beam radiography.	Expansion through the use of expander anchored on miniscrews allowed an increase in maxillary suture more than 2.5 times compared to expansion with conventional rep.
Halicioglu et al. (2016) [43]	Randomized controlled trial	Examine and contrast the effects of a memory screw versus a traditional HE screw on the soft tissues and skeletal features of the face.	32 patients divided into 2 groups: 17 treated with memory screw palatal expander and 15 patients treated with Hyrax screw expander.	Mid-palatal suture opening was obtained in all patients. Anterior and inferior rotation of the upper jaw was observed in both groups; instead, the jaw rotates downward and backward especially in the memory-screw group.
Manzella et al. (2021) [44]	Retrospective controlled clinical study	In adolescent orthodontic patients who have maxillary transverse constriction to assess the dental effects of the Ni-Ti Memoria^®^ Leaf Spring Activated Expander (MLSAE)	The sample consisted of 22 patients who received Ni-Ti MLSAE treatments in a row and 22 untreated controls. Digital dental casts were obtained at baseline, 7 days, 4 weeks, and after jaw expansion. Misure of inter-canine, inter-premolar, inter-first molar area, depth and perimeter of arch, and angulation of molars measurements of the maxillary dental arch were assessed.	Ni-Ti MLSAE can achieve sufficient expansion without significantly tipping the patient’s teeth.
Ugolini et al. (2020) [20]	Randomized trial	Analysis how two different type of palatal expansion screws influenced function throughout the first 7 days of activation.	101 individuals were randomly allocated to one of two groups: (1) those who received the Hyrax screw; (2) those who received the LE. A comparison was made between the two groups regarding reported pain.	Clinical activation protocol and screw type have an impact on pain experienced during maxillary arch expansion. Patients who utilized a LE reported much decreased discomfort throughout the first week of therapy.
Lanteri et al. (2020) [45]	Clinical trial	After maxillary expansion using a slow maxillary expansion protocol, changes in vestibular bone thickness (BT) and dento-alveolar buccal tipping of first and second molars were investigated.	The Leaf Expander protocol was used to treat 20 patients. Cone beam computed tomography (CBCT) images were used to calculate the buccal alveolar BT, buccal alveolar bone height (BH), dental TIP, and inter-molar width (IW) regarding the first and second primary molars.	After using LE appliance, it seems that BT was not substantially decreased. During mixed dentition, the treatment of maxillary hypoplasia through a slow expansion with Ni-Ti springs looks successful and secure.
Serafin et al. (2022) [46]	Randomized clinical trial	To compare skeletal and dental changes following Leaf Expander (LE)-performed rapid maxillary expansion (RME) and slow maxillary expansion (SME).	Patients treated with palatal expanders anchored on deciduous second molars were divided into 2 groups: (1) RME group (16 patients treated with Hyrax expander); (2) SME group (16 patients treated with LE). Then, they made a comparison of the effects of these appliances.	Both RME and SME effectively expanded the skeleton and the dentoalveolar region; RME did so more anteriorly than SME, but with less control over the development of permanent molar decompensation. SME by LE could, therefore, be a useful and effective alternative in patients who are growing.
Davami et al. (2020) [47]	Randomized clinical trial	Evaluate and compare, with CBCT, the skeletal and dental alterations following maxillary expansion through a tooth-supported expander and with a bone-supported expander.	29 young patients were separated into two groups: (1) bone supported expander; (2) tooth supported expander. Before and after the expansion CBCT was taken.	Expansive results are superimposable with both types of expansion
Lanteri et al. (2018) [25]	Pilot study	Study of the dentoalveolar effects of SME using the LE in growing patients with deficit of transverse diameter of maxilla.	10 patients with mixed dentition were treated with LE. 5 factors were considered: (1) the distance between the first upper permanent molars; (2) the distance between the second upper deciduous molars; (3) the distance between the cusps of the upper canines; (4) the distance between the lower first permanent molars; (5) the distance of the lower canine cusps.	The LE guarantees good results in the correction of transverse maxillary deficit in mixed dentition.
Silveira et al. (2021) [48]	Randomized controlled clinical trial	Comparing the Hyrax and Mini Hyrax’s impacts on growing patients’ oral health, quality of life, and pain perception.	34 boys with transverse maxillary deficiency were divided into 2 groups, who had Mini Hyrax expander (MHE) and who had Hyrax expander (HE). Dental impacts were measured using digital overlays and variations in quality of life were measured using a questionnaire.	The dental outcomes, quality of life impacts or pain perception of teenagers wearing the Hyrax Mini (MHE) and Hyrax Expanders (HE) did not differ significantly.
Cossellu et al. (2020) [49]	Clinical study	Evaluate the effects of SME on the maxillary and mandibular arch, using LE appliance inserted on the second primary molar.	90 patients with posterior crossbite and transverse maxillary deficiency and were divided into 69 undergoing SME and 21 treated with RME.	The effectiveness of SME has been confirmed on both the upper jaw and mandibular arches.
Feldmann et al. (2017) [50]	Randomized controlled trial	Assess the level of pain, degree of discomfort, and jaw function impairment during the first week with RME	54 patients divided in different groups: (A) Conventional Hyrax Group (HG), (B) Patients received a HHA attached to anterior palate mini-implants. On the 1st and 4th days following RME appliance insertion, questionnaires were utilized to measure pain severity, discomfort, analgesic intake, and impairment of jaw function.	During the first week of treatment, tooth and bone expansion was generally well accepted by patients and regardless of gender.

**Table 3:** Pain level and pain index for Hyrax and Leaf expander, respectively.

Device	Pain Level	Pain Index
Hyrax	88.6%	51.4%
Leaf expander	25%	9.7%

## Data Availability

Not applicable.

## Abbreviations

BT	Buccal alveolar bone thickness
BH	Buccal alveolar bone height
CBCT	Cone beam computed tomography
RPE	Rapid palatal expander
MLSAE	Leaf spring activated expander
MARPE	Miniscrew assisted rapid palatal expansion
RME	Rapid maxillary expansion
SME	Slow maxillary expansion
LE	Leaf expander
HHA	Hybrid Hyrax Appliance
HHG	Hybrid Hyrax group
HG	Conventional Hyrax group
IW	Inter-molar width
MSG	Memory screw group
MS	Memory screw
HSG	Hyrax screw group
TAD’s	temporary anchorage device
TMJ	temporary mandibular joint
